# NanoTube Construct: A web tool for the digital construction of nanotubes of single-layer materials and the calculation of their atomistic descriptors powered by Enalos Cloud Platform

**DOI:** 10.1016/j.csbj.2024.09.023

**Published:** 2024-10-05

**Authors:** Panagiotis D. Kolokathis, Dimitrios Zouraris, Nikolaos K. Sidiropoulos, Andreas Tsoumanis, Georgia Melagraki, Iseult Lynch, Antreas Afantitis

**Affiliations:** a NovaMechanics MIKE, Piraeus 18545, Greece; b NovaMechanics Ltd, Nicosia 1070, Cyprus; c Entelos Institute, Larnaca 6059, Cyprus; d Division of Physical Sciences and Applications, Hellenic Military Academy, Vari 16672, Greece; e School of Geography, Earth and Environmental Sciences, University of Birmingham, Birmingham B152TT, United Kingdom

**Keywords:** Nanotube Construction, Single-layer Materials, Energy Minimization, Graphane, MoS₂, Graphyne, Nanotube, Nanosheet, Nanotube Builder

## Abstract

NanoTube Construct is a web tool for the digital construction of nanotubes based on real and hypothetical single-layer materials including carbon-based materials such as graphene, graphane, graphyne polymorphs, graphidiyene and non-carbon materials such as silicene, germanene, boron nitride, hexagonal bilayer silica, haeckelite silica, molybdene disulfide and tungsten disulfide. Contrary to other available tools, NanoTube Construct has the following features: a) it is not limited to zero thickness materials with specific symmetry, b) it applies energy minimisation to the geometrically constructed Nanotubes to generate realistic ones, c) it derives atomistic descriptors (e.g., the average potential energy per atom, the average coordination number, etc.), d) it provides the primitive unit cell of the constructed Nanotube which corresponds to the selected rolling vector (i.e., the direction in which the starting nanosheet is rolled to form a tube), e) it calculates whether the Nanotube or its corresponding nanosheet is more energetically stable and f) it allows negative chirality indexes. Application of NanoTube Construct for the construction of energy minimised graphane and molybdenum disulfide nanotubes are presented, showcasing the tool's capability. NanoTube Construct is freely accessible through the Enalos Cloud Platform (https://enaloscloud.novamechanics.com/diagonal/nanotube/).

## Graphical Abstract

**Figure:**
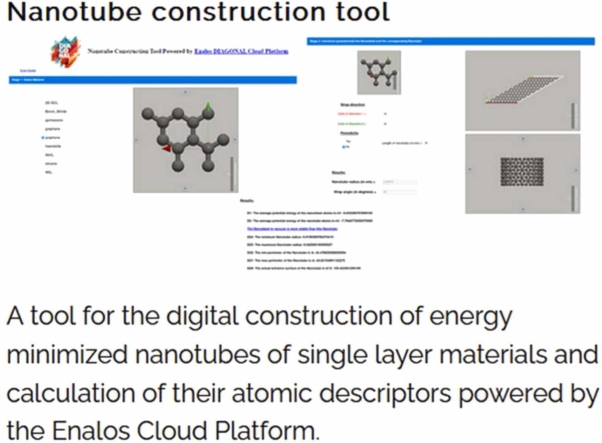

## 1 Introduction

In recent years, the development of nanomaterials (NMs) has increased remarkably due to their distinct physical, chemical, and mechanical properties which differ significantly from the respective bulk material properties [1], [2]. Their exceptional properties, including high surface-to-volume ratio, enhanced catalytic activity, and unique quantum mechanical behaviours, demand detailed studies to fully exploit their potential and ensure that they can be utilised safely and sustainably.

To meet this demand, computational approaches have become crucial in investigating and designing NMs and to shed light on stable structures that may have not been synthesised as yet (e.g., nanotubes which are the structures investigated in this work). Techniques like density functional theory (DFT) and molecular dynamics (MD) simulations provide insights into the atomic and molecular interactions governing NMs' (including nanotubes) behaviour [3], [4]. These methods are less time-consuming than experimental techniques while they provide precise predictions of material properties which makes them appropriate for material screening for specific properties and applications. Consequently, these computational techniques are vital to the Safe and Sustainable by Design (SSbD) framework, helping researchers predict environmental and health impacts and guiding the development of safer, more sustainable materials [5], [6].

Due to their properties, single-layer materials are considered to be promising for numerous applications in catalysis, sensing and energy storage, and as such represent excellent candidates for demonstration of the utility of the SSbD framework and its application at the early stages of materials design. For instance, graphene, the most well-known single-layer material, exhibits remarkable electrical conductivity, mechanical strength, and thermal conductivity due to its two-dimensional structure and zero thickness of its atomic layer [7], [8]. The extensive surface area of single layer materials allows for numerous active sites for chemical reactions or adsorption processes, enhancing performance in these applications [9], [10].

Single-layer materials can form various structures, such as sheets and nanotubes, influenced by their atomic and molecular interactions. For example, graphene can form carbon nanotubes (CNTs) when a graphene sheet is rolled up. This ability to form tubular structures depends on the material's intrinsic properties, such as atomic arrangement and energy barriers influencing stability. Numerous attempts have been made to predict the structure and properties of nanotubes using computational tools like DFT and MD simulations. The initial nanotube configurations needed can be produced by currently available tools (e.g., VMD Nanotube Plugin [11], Wolfram Nanotube Builder [12], TubeGen [13], Nanotube Modeler [14]). However, these tools typically focus on materials with hexagonal symmetry and zero thickness [8], [10], [15] and they do not apply energy minimization to get more realistic structures than the geometrically generated ones. To the best of our knowledge, there is no tool to digitally construct energy minimised graphene [16], [17], graphyne [18], graphidyene [19], haeckelite silica [20], [21], hexagonal bilayer silica [20], [22], molybdene disulfide [23], [24], and tungsten disulfide [25], [26], [27] nanotubes. From the aforementioned materials, graphane, molybdene disulfide, and tungsten disulfide nanotubes have already been synthesised. Graphane nanotubes can be synthesised through a full hydrogenation of carbon nanotubes [28], [29], tungsten disulfide nanotubes can be synthesized by reaction of tungsten oxide with H_2_S and H_2_ gases [26] and molybdene disulfide nanotubes can be synthesised by molybdene disulfide deposition on single wall carbon nanotubes by chemical vapor deposition [24]. Graphyne, graphidyene, haeckelite silica and the hexagonal bilayer silica have been synthesised as nanosheets [20], [30], [31] but there is no synthesis procedure for their corresponding nanotubes to the best of our knowledge. NanoTube Construct aspires to reveal whether these materials can exist in the nanotube form or not and to calculate computationally their properties providing extra motivation for their synthesis in case these properties satisfy the requirements for specific applications.

Another available tool for the geometrical construction of nanotubes of non-zero thickness single layer materials, Chiraltube [32] has been developed in the same period of time with Nanotube Construct [33], [34]. However, Chiraltube [32] is limited to the geometrical construction of nanotubes with non-negative chirality indexes while Nanotube Construct allows the selection of negative chirality indexes and produces energy minimized nanotubes which are more realistic due to the consideration of the effect of Force-Fields on Nanotube atoms. Negative chirality indexes are necessary to define all possible nanotubes for structures with low symmetry in case there is no pair of positive indexes that correspond to a pair having a negative chirality index. For example, the pairs of positive chirality indexes can fully define nanotubes of high symmetry materials such as graphene because there is always a positive pair of chirality indexes that corresponds to a pair having a negative chirality index. However, this is not the general case, and negative chirality indexes are required. The definition of the chirality indexes for carbon nanotubes can be found in the work of Dresselhaus et al. [35].

Because the definition of the chirality indexes is highly related to the selection of the unit cell and its unit cell vectors, NanoTube Construct illustrates the unit cell of the material including the unit cell vectors and the positions of atoms. Absence of this information (e.g., selection of a single-layer material from a list without the previously mentioned additional information) leads to ambiguous definitions of the chirality index among tools. This happens because the selection of different unit cell vectors could lead to different chiral indexes for the same nanotube. In addition to the previously mentioned tools, NanoTube Construct compares the stability of a nanosheet with the constructed nanotube by predicting the possibility of nanosheet rolling and derives atomistic descriptors that can be used to enrich an experimental dataset for the development of a Machine Learning model for the prediction of toxicity similar to other works in the past [36].

Besides the chirality indexes and the unit cell information, the rolling of the sheet (i.e., selection of the one of the two surfaces of the sheet that will be the interior surface of the nanotube) is needed to fully describe a nanotube. Despite this is not necessary to fully describe nanotubes of zero thickness materials such as graphene, the selection of different rolling surface for these materials when the chiral indexes are equal leads to chiral nanotubes. On the other hand, the information about the rolling of the sheet is necessary to fully define nanotubes of non-zero thickness materials with low symmetry (i.e., there is no symmetry along the axis vertical to the surface of the sheet). In case of symmetry along the axis vertical to the sheet, the two surfaces of the sheet have exactly the same properties, and there is no need for such a distinction. Until now, NanoTube Construct’s list of materials does not include non-zero thickness materials with absence of symmetry along the axis vertical to the surface of the sheet, and consequently the parameter of the rolling of the sheet is not mentioned. As the list of the materials in NanoTube Construct will continuously increase during time by the entrance of new materials, the rolling of the sheet will be included as a parameter in case of the entrance in the list of a material with non-zero thickness and no symmetry along the axis vertical to the surface of the sheet.

Among the list of the single-layer materials available in Nanotube Construct are graphynes. Graphyne structures were predicted theoretically by Baughman et al. [18] who showed that its formation energy is much lower than other carbon phases as a result of graphyne having acetylenic groups (i.e., doubly unsaturated positions or C-C triple bonds (*sp*-hybridized) on a molecular framework) as major components. This was the motivation for synthesis of graphyne structures [30], [31]. The distances between different types of carbons (sp, sp^2^) proposed by Baughman et al. [18] were used to digitally construct the primitive unit cells of graphyne materials available in Nanotube Construct. According to Baughman *et al.* [18], a wide range of graphyne structures can be constructed by different arrangements of the different carbon types having a different ratio of sp and sp^2^ carbons. To distinguish these structures, Baughman et al. [18] proposed a specific notation with three different indexes (*x*, *y*, *z*) where *x* is the number of carbon atoms in the smallest ring (1 R) of the structure, *y* is the number of carbon atoms in the next smallest ring (2 R) which is interconnected with the smallest one (1 R) via a C(sp^2^)C(sp)C(sp)C(sp^2^) rod and *z* is the number of atoms in a third ring connected to the 2 R ring by a C(sp^2^)C(sp)C(sp)C(sp^2^) rod. For convenience, the notation of *α*, *β* and *γ* graphynes is used here instead of the indexes (18, 18, 18), (12, 12, 12) and (6, 6, 6) respectively [37], [38].

**Fig. 1:**
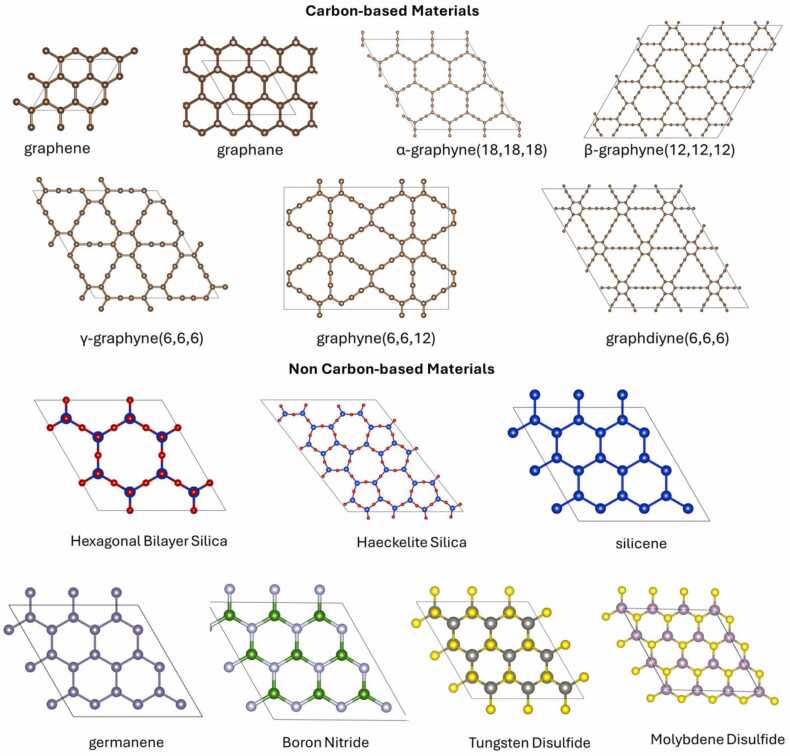
Single layer Materials options available in Nanotube Construct. Carbon, hydrogen, silicon, oxygen, germanium, boron, nitrogen, tungsten, sulphide and molybdenum atoms are illustrated with brown, pink, bright blue, red, dark blue, green, light blue, dark grey, yellow and light magenta respectively.

Starting from any graphyne structure and replacing each one of its acetylenic carbon-carbon triple bonds (‘‘–C≡C–’’ linkages) with a diacetylenic ‘‘–C≡C–C≡C-’’ linkage, we get Graphdiyne [19], [38], [39], [40]. Generalising the process by replacing any acetylenic linkages of graphyne with an *n*-acetylenic ‘‘(–C≡C–)_n_’’ linkage, we get the structure of graphyne-n [39]. From the graphdiynes family, the γ-graphdiyne [40] (or the graphdiyne (6,6,6) polymorph according to the other notation) was selected to be in the list of the materials used by NanoTube Construct to build the γ-graphdiyne nanotube. Although γ-graphdiyne has already been synthesised [40], its corresponding nanotubes have not been synthesised as yet to the best of our knowledge. NanoTube Construct can be used to predict which rolling direction of the γ-graphdiyne sheet leads to the most stable nanotube (see later for more details of how this is determined via Nanotube Construct). In addition to these materials, NanoTube Construct can build nanotubes by rolling silicene [41], [42], germanene [41], [42] (i.e., Group-IV monolayer materials) and boron nitride sheets. In contrast to graphene, where the chemical bonds between neighbouring atoms are strong enough to keep its structure planar [35] which means that the resulting tubes have zero thickness, silicene and germanene have a thickness of 0.46 Å and 0.64 Å, respectively, which is the height difference between a silicon or germanium atom and its neighbouring silicon or germanium atoms [43]. The structures of the aforementioned material structures (i.e., materials options of Nanotube Construct) are illustrated in Fig. 1.

By selecting different combinations of sp and sp² hybridised carbon atoms in graphyne, graphdiyne and graphyne-*n* materials, we can tune the band gaps and the electron mobility, making materials suitable for specific electronic and photonic applications. These properties also change after the wrapping of these nanosheets to create nanotubes. The direction of the rolling can also be used to tune these properties and the NanoTube Construct application can be used to make these structures which can then be used for Density Functional Theory Calculations to investigate the materials’ electronic and photonic properties [42]. Another single-layer material that has been included in the list of NanoTube Construct material options, and which has been synthesized successfully in its nanotube form, is Boron Nitride [44]. Boron Nitride nanotube is an electrical insulator which is also used as a protective shield for various NMs [44].

To avoid the requirement that the user needs a full theoretical understand of the scientific computations applied during the sheet rolling (=straight line becomes an arc) and energy minimization processes applied before and after rolling, NanoTube Construct comes with a Graphical User Interface (GUI), and it is easily usable by a broad range of researchers. It can be integrated with other predictive models already available on the cloud platform, which is crucial for designing nanotubes with specific biological and chemical properties and as part of the safe and sustainable by design approach. Additionally, NanoTube Construct provides critical atomistic descriptors for the nanosheets and associated nanotubes as input values for machine learning models, facilitating advanced data analysis and property optimization. Employing the Software as a Service (SaaS) model, NanoTube Construct is accessible and scalable, requiring no complex installations and it offers significant advantages in material diversity, integration, and ease of use, positioning it as an essential tool for advancing NMs research.

NanoTube Construct aims to reduce the time-consuming trial-and-error experiments currently applied in materials discovery with theoretical calculations [45] by calculating the atomistic descriptors needed as input to Machine Learning models and helps in the design of safer and more effective materials [46].

To demonstrate the capabilities and the use of Nanotube Construct, two examples are presented in this paper concerning the stability of the nanotubes, one from a carbon-based and one from a non-carbon-based material, namely graphane and molybdene disulfide, while the hexagonal bilayer silica [20] has been selected to demonstrate how to use the Nanotube Construct’s GUI (see next section). Graphane was chosen due to the growing interest in this material and its potential applications. Graphane's unique physical, chemical, and mechanical properties, including its insulating nature, mechanical strength, and thermal stability, make it a promising material for advanced applications [16], [47]. Its structure - a single layer of carbon atoms in a hexagonal lattice that is fully hydrogenated on both sides - makes it appropriate for applications such as hydrogen storage, flexible electronics, and nanocomposites. Furthermore, graphane's two-dimensional nature allows further functionalization by replacement of its hydrogen atoms, facilitating the creation of new nanostructures, which could be suitable for a wide range of applications [48], [49], [50].

Among the non-carbon materials, molybdenum disulfide (MoS₂) is a layered transition metal dichalcogenide (TMD) known for its unique properties and applications in electronics, optoelectronics, and energy storage. Its sandwich-type S-Mo-S hexagonal structure offers high electron mobility, mechanical flexibility, and a direct bandgap in its monolayer form, making it a promising material for next-generation devices [51], [52]. MoS₂ nanotubes can be synthesised using methods like chemical vapour deposition and sulphiation of oxides, allowing precise control over their structural and electronic properties [51], [53]. Depending on their chirality, armchair MoS₂ nanotubes are indirect bandgap semiconductors, while zigzag nanotubes are direct bandgap semiconductors, influencing their use in nanoelectronics and optoelectronics [52]. MoS₂ nanotubes also exhibit high tensile strength and flexibility, essential for flexible electronic devices such as wearable devices [54], while computational methods like density functional optoelectronics (molecular dynamics, MD) simulations have been pivotal in understanding MoS₂ nanotubes [51], [52], [55].

## 2 Description of NanoTube’s Graphical User Interface and its integration into the Enalos Cloud Platform

NanoTube Construct is divided into three consecutive Stages, with completion of each stage being the pre-requisite to progression to the next stage. The derived output files can be downloaded after the end of the second or the third Stages.

**Stage 1: Selection of single layer material and visualization of its unit cell**

**Fig. 2:**
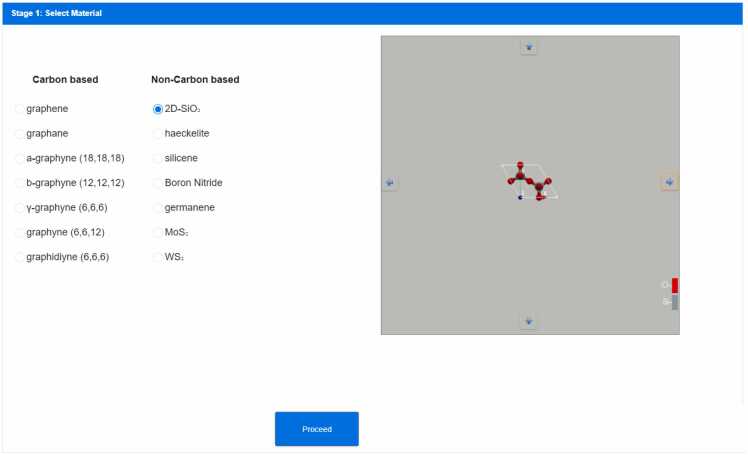
Selection of a single layer material for the construction of a nanotube from a predefined list of single layer materials. The unit cell of the selected material appears on the right after the user presses the proceed button. The blue arrows can be used to rotate the unit cell.

Stage 1 includes the selection of a single-layer material from a predefined list. Upon selection, the primitive unit cell of the chosen material is displayed (see Fig. 2). In our first demonstration case, the hexagonal bilayer silica [20] has been selected from the list of available materials included in NanoTube Construct to demonstrate how the NanoTube Construct GUI can be used to build an energy minimised Nanotube having first selected the specific rolling direction of its nanosheet (see Stage 2). The rotation of the unit cell is available through the blue arrows of the NanoTube Construct GUI (see Fig. 2) and helps the user to select the rolling direction that will be applied to the sheet to make its corresponding Nanotube.

**Stage 2: Geometrical digital construction of nanotube by rolling the sheet/unit cells of Stage 1 in a direction selected by the user**.

**Fig. 3:**
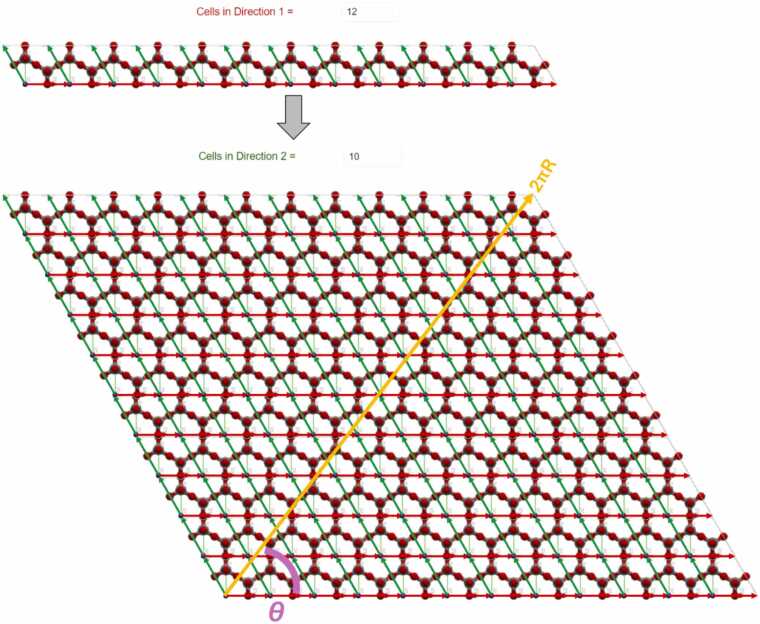
Replication of the unit cell for hexagonal bilayer silica defined in Stage 1. Top: The unit cell is replicated 12 times in direction 1 (red vectors) and the row is then replicated in direction 2 (green vectors) 10 times (bottom). The rolling vector (orange colour) illustrates the rolling direction and its length equals to the perimeter of a cylinder of Radius R and thus defines the width of the nanotube.

**Fig. 4:**
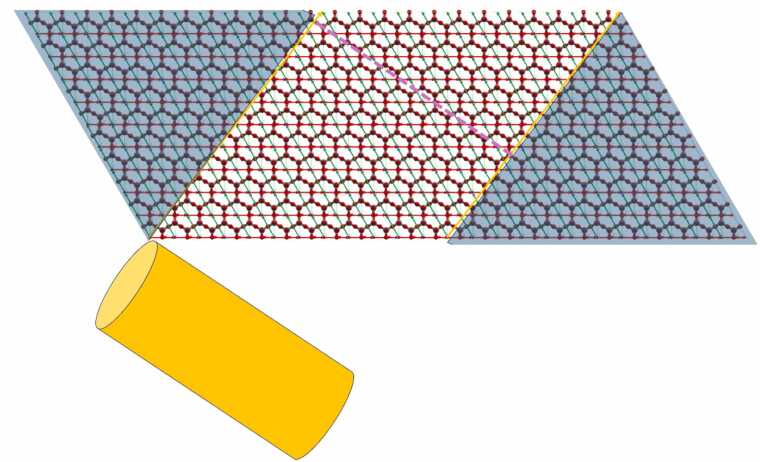
Digital Construction of the Nanosheet required to build the Nanotube by rolling. The pink dashed line indicates the length of the resulting nanotube (which is shown below the sheet in orange). The shaded parts outside the rolling vectors are discarded from the simulation.

**Fig. 5:**
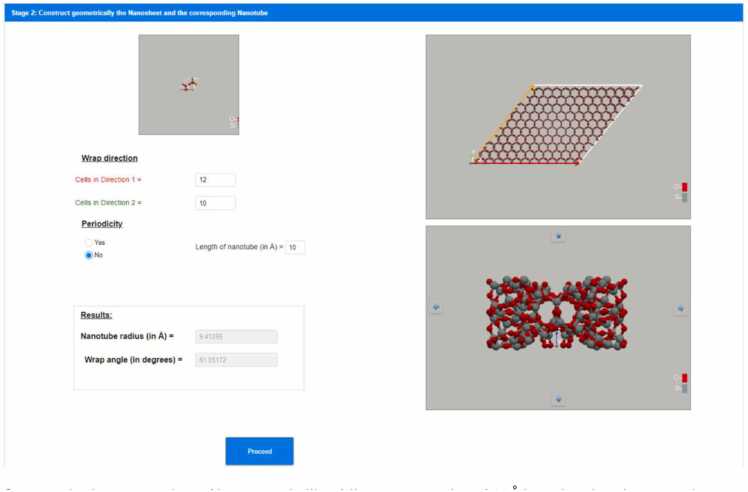
Geometrical construction of hexagonal silica bilayer nanotube of 10 Å length using the wrapping vector (orange lines) of Figure.

**Fig. 6:**
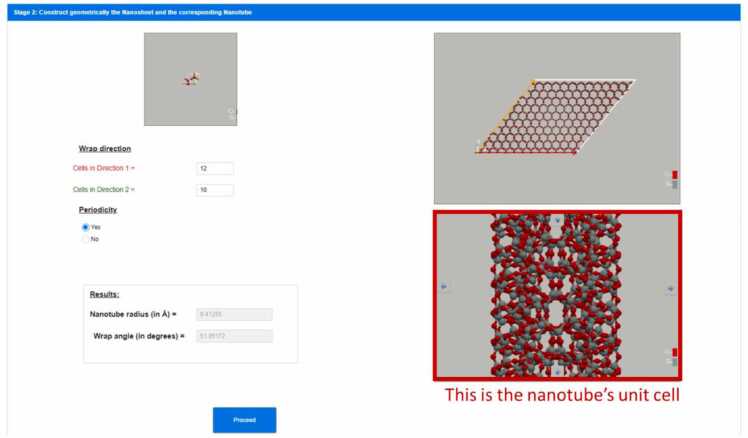
Geometrical construction of hexagonal silica bilayer nanotube unit cell using the wrapping vector of Figure (orange line).

To make the nanotube, the rolling direction/vector should be selected and the nanosheet that will be rolled should be constructed. To select the rolling direction/vector the user inserts the replication number per direction 1 and 2 (width and length, respectively) of the unit cell defined in Fig. 2. By replicating the unit cell, the unit cell vectors are also replicated. The unit cell vectors are the green and red vectors of Fig. 3 and they are the unit cell vectors for directions 1 and 2. The vector sum of all of the unit cell vectors of the replicated unit cells is the rolling vector which defines the rolling direction (see orange vector in Fig. 3) and consequently, the notation (*n*_1_, *n*_2_) where *n*_1_ and *n*_2_ are the replication numbers per directions 1 and 2, has been selected to describe the rolling vector. Positive and negative integer values are allowed to be used. Negative direction means replication in the opposite direction than the direction of the unit cell vector (see green and red vectors of Fig. 3 which are the unit cell vectors for direction 1 and 2). A replication number can be zero in one direction only, otherwise it is not a vector but a point. The replication of the unit cell is described in Fig. 3 where a nanosheet is created. However, this nanosheet needs further processing to be used for the nanotube construction via Nanotube Construct. This process is described in Fig. 4, whereby the nanosheet of Fig. 3 is replicated twice in direction 1 and we keep only the area between the two rolling vectors, i.e., the orange vectors shown in Fig. 4. This area includes the nanosheet that if it is rolled creates the target Nanotube. Fig. 3 shows that the length of the rolling vector determines the radius of the nanotube while its direction determines the wrap/rolling angle. Despite a nanotube being constructed using this procedure, this does not assure that it can be replicated in z direction to make longer and longer nanotubes due to mismatch of the top and bottom ends of the nanotube. This is only possible for nanotubes that have specific length which satisfies the periodic boundary conditions. The box containing this nanotube is called the primitive unit cell of the nanotube and it can be used further to build longer nanotubes while it can be used to simulate infinite length nanotubes by applying periodic boundary conditions. The user can select the construction of a non-periodic and specific length nanotube (see Fig. 5) or the construction of the unit cell of the nanotube (see Fig. 6). Consequently, each rolling vector defines a nanotube and each of these nanotubes has its unique primitive unit cell. If there is a perfect match of the nanotube at the top and bottom of the pink line shown in Fig. 4, then the nanotube will satisfy periodicity and can be used to make its primaitive unit cell. Otherwise, the sheet of Fig. 4 should be replicated as many times as needed so that there is less than a threshold value difference at the top and the bottom of the nanotube. The selected threshold in NanoTube Construct is 0.0001 Å. After the creation of the Nanotube, the wrap angle (see Fig. 3) as well as the Nanotube radius are printed in the GUI.

**Stage 3: Digital construction of energy minimised nanotube after applying energy minimisation to the nanotube created in stage 2 and calculation of its atomistic descriptors**

**Fig. 7:**
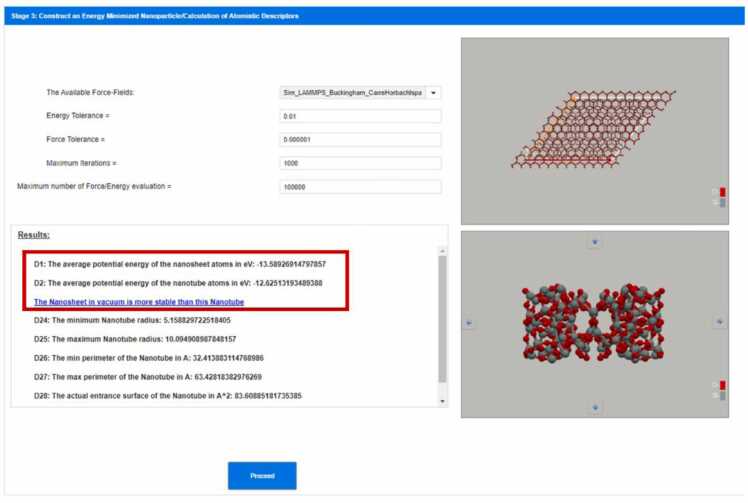
Energy minimized hexagonal silica bilayer nanosheet (top) and nanotube (bottom) and calculation of their atomistic descriptors.

After the creation of the geometrically constructed nanotube, energy minimization should be applied to get a realistic structure of the nanotube. To do this, a force-field should be selected. NanoTube Construct collects the available Force-Fields that contain parameters for every chemical element that exists in the nanotube from the OPENKIM database (Fig. 7) by following the strategy described in our previous work [56], [57]. These force-fields appear in a dropdown list starting from the more specific (i.e., applicable to less chemical elements) to the more generic (i.e., applicable to a wider range of chemical elements) Force-Fields. This step is critical for ensuring the structural stability of the nanotube. By the end of the energy minimization process the average potential energy per atom has been calculated and serves as a key indicator of the nanotube's stability. All the calculations performed by NanoTube Construct refer to Nanotube and Nanosheets in vacuum and may differ from the calculations performed in the presence of a solvent. Comparing the calculated average potential energy per atom in the nanotube value with that of the sheet helps to determine the relative stability of both structures (see Fig. 7). A small difference suggests that both configurations are similarly stable, guiding synthetic chemists in experimental synthesis. Additional atomistic descriptors, such as the average coordination number (see Refs. [56], [57]), can be utilised as inputs for machine learning models to predict other properties, such as toxicity or reactivity, enhancing the material design process.

**Fig. 8:**
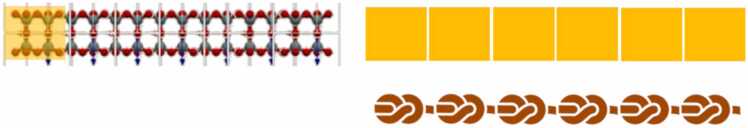
Replication of hexagonal silica bilayer unit cell in space, and the creation of a “chain” (left). A sketch of this chain by simplified rectangular shapes replacing the unit cells is also shown (right).

**Fig. 9:**
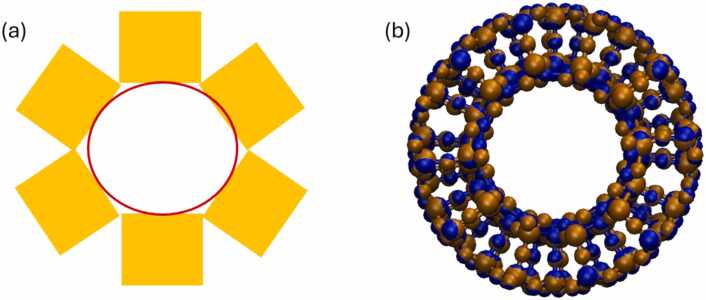
(a) Projection of Cylinder (red colour) inscribed inside the chain of the unit cells (yellow colour). The effect of the rolling on the innermost atoms can also be visualised from this schematic, (b) Geometrically Constructed (Blue) and Energy minimized (Brown) hexagonal silica bilayers Nanotube atoms with (12,10) chiral indexes created after applying the procedure described in Fig. 6, Fig. 7.

The difference in stability of a nanotube and nanosheet can be explained by the counteraction of the repulsion due to the deformation of the sheet and the attraction created by the increase of van der Waals forces due to the increase in the coordination number (i.e., neighbouring atoms) upon rolling. To better understand the deformation that the unit cell experiences during the nanotube construction process, we start with the creation of a chain of unit cells (i.e., in our example the hexagonal bilayer silica), by replicating six times in *x* direction (see Fig. 8). The edges of the chain can be linked due to the property of continuity/periodicity of their unit cell to make a closed chain inside of which a cylinder is inscribed (see the projection of the cylinder in Fig. 9). Fig. 9 shows that there is not a perfect match of the unit cells in the outer surface of the nanotube because the edges of the unit cells do not coincide in the outer surface which means that they need to be distorted to do so. Fig. 9 shows that the interatomic distances in nanotube/cylinder have increased compared to the distance of the linear chain of Fig. 8. This interatomic distance increase is larger for the outer atoms of the unit cell (see Fig. 9) than the inner ones, and it increases more the greater the unit cell’s thickness. Consequently, a geometrical construction of a nanotube may not lead to realistic structures and energy minimization must be applied in order to get a stable nanotube structure (see Fig. 9-b). During energy minimization, the nanotube's internal radius (i.e., the cylinder radius of Fig. 9 can be reduced, stay the same or be increased because of the exerted forces. As we have discussed in previous work [56], [57], the nanotube can be trapped in a local minimum and an atomistic simulation at higher temperature may be necessary to overcome this local minimum to reach the global minimum. To do this, the LAMMPS datafiles for the created structures are provided by pressing the “Download the Output Files” button. The properties of the constructed nanotube and the nanosheet, such as nanotube surface, radius, etc., before and after energy minimization can be useful as atomistic descriptors to enrich the dataset for subsequent Machine Learning models [36]. Note that starting with a cylindrical configuration does not necessarily mean that the nanotube will remain cylindrical, but may acquire ellipsoid characteristics.

## 3 Description/definitions of the calculated descriptors

The calculation of atomistic descriptors (D) is crucial for understanding the properties of NMs [36], [56], [57]. These descriptors offer detailed insights into the structural and energetic characteristics of the materials, which are essential for predicting their behaviour and optimising their applications. The average potential energy of the nanosheet atoms (Descriptor 1 (D1) in Fig. 7) quantifies the mean potential energy per atom within the nanosheet structure, serving as an indicator of overall stability and reactivity, with lower values denoting more stable configurations. Similarly, the average potential energy of the nanotube atoms (D2 in Fig. 7) measures the stability of the nanotube, with lower values indicating greater stability compared to the nanosheet. The stability comparison reveals that the nanotube in vacuum is more stable than the nanosheet, as derived from the lower potential energy of the nanotube. The minimum nanotube radius (D24) and maximum nanotube radius (D25) provide insights into the geometric constraints and uniformity of the nanotube's dimensions, which are vital for precise applications in nanotechnology. The minimum perimeter (D26) and maximum perimeter (D27) of the nanotube's cross-section influence the surface area and interaction sites, impacting its chemical and physical properties. The actual entrance surface at the open ends of the nanotube (D28) quantifies the surface area at the nanotube entrance, crucial for applications involving fluid dynamics, molecular transport, and surface reactions within the nanotube. Finally, the nanotube thickness (D29) measures the wall thickness, determining the mechanical strength, flexibility, and overall durability of the nanotube. These atomistic descriptors provide a comprehensive understanding of the structural and energetic properties of both nanosheets and nanotubes, facilitating the design, optimization, and application of NMs in various advanced technological fields. We can see the whole list of descriptors in Table 1.

**Table 1:** List of descriptors calculated by Nanotube Construct.

D1: The average potential energy of the nanosheet atoms in eV
D2: The average potential energy of the nanotube atoms in eV
D3: The average difference of the potential energy between nanosheet and nanotube atoms in eV
D4: The average ratio of the potential energy between nanosheet and nanotube atoms
D5: The average coordination parameter of the nanosheet atoms
D6: The average coordination parameter of the nanotube atoms
D7: The average difference of the coordination parameter between nanosheet and nanotube atoms
D8: The average ratio of the coordination parameter between nanosheet and nanotube atoms
D9: The diameter of the Nanotube in Å
D10: The perimeter of the Nanotube in Å
D11: The entrance surface of the Nanotube in Å^2^
D12: Unit Cells to replicate for wrap in direction 1 (n_1_)
D13: Unit Cells to replicate for wrap in direction 2 (n_2_)
D14: Absolute difference 0 f (n_1_) - (n_2_)
D15: n_2_ / n_1_
D16: The average CNP of the nanosheet atoms
D17: The average CNP of of the nanotube atoms
D18: The average difference of the CNP between nanosheet and nanotube atoms
D19: The average ratio of the CNP between nanosheet and nanotube atoms
D20: The average first hex parameter of the nanosheet atoms
D21: The average first hex parameter of the nanotube atoms
D22: The average second hex parameter of the nanosheet atoms
D23: The average second hex parameter of the nanotube atoms
D24: The minimum Nanotube radius in Å
D25: The maximum Nanotube radius in Å
D26: The min perimeter of the Nanotube in Å
D27: The max perimeter of the Nanotube in Å
D28: The actual entrance surface of the Nanotube in Å^2^
D29: The Nanotube thickness in Å
D30: The Nanosheet thickness in Å

## 4 Results and discussion

As a first example to showcase the use of the NanoTube Construct, graphane was chosen due to the growing interest in its potential applications. The geometrical construction of the nanosheet and the corresponding nanotube using the NanoTube Construct begins with initialising a single-layer graphane sheet, characterised by its hexagonal lattice structure of carbon atoms. The next step involves setting the rolling directions by specifying the number of unit cells in two directions, Direction 1 and Direction 2 (see the description of Stage 2 above). Various configurations are explored, such as rolling vectors (10, 10), (20, 10), (30,10), (40, 10), (50,10), (60, 10), (70, 10), (80, 10), (90, 10) and (100, 10) (see Fig. 2, Fig. 3, to observe how the rolling vector affects the nanotube's geometry and the description of Stage 2 above for its notation). The periodicity option is selected to make the primitive unit cell of the nanotube (see the description of Stage 2 above) which is used to calculate the properties/descriptors for an infinite length nanotube. The tool then wraps the graphane sheet along the specified directions, and the resulting structure is visualised in 3D. The tool calculates the nanotube's geometrical parameters, such as the radius and wrap angle (Table 2). Observations reveal that increasing the number of cells in Direction 1 increases the nanotube radius from approximately 2.44 Å to 36.61 Å, while the wrap angle decreases from 60 degrees to 5.21 degrees, resulting in a more extended nanotube. Choosing the periodicity option ensures that the resulting nanotube structure is stable and accurately represents a continuous cylinder, which is essential for realistic simulations and material studies.

**Table 2:** Geometrical parameters of graphane nanotubes constructed using the NanoTube Construct Tool: Number of cells in Direction1 and Direction 2, nanotube radius and wrap angle.

Cells in Direction 1	Cells in Direction 2	NanoTube Radius (Å)	Wrap Angle (Degrees)
10	10	2.44	60.00
20	10	5.36	30.00
30	10	9.00	19.11
40	10	12.85	13.90
50	10	16.76	10.89
60	10	20.71	8.95
70	10	24.67	7.59
80	10	28.62	6.59
90	10	32.61	5.82
100	10	36.61	5.21

In the process of constructing the nanotube using NanoTube Construct, only the number of cells in Direction 1 was varied while keeping the number of cells in Direction 2 constant. Due to technical reasons (i.e., memory demands, computational time), NanoTube Construct is limited to less than 100 cells per direction. By keeping the number of unit cell replications in Direction 2 constant, the study can focus on exploring the effects of varying the number of unit cell replications in Direction 1 on the resulting nanotube's properties, such as its radius and wrap angle. By increasing the number of unit cell replications in Direction 1, the circumference of the nanotube increases, which directly influences the nanotube's radius. This allows for a clear observation of how changes in the graphane rolling vector affect the overall size, the geometry and the properties of the resulting nanotube.

Then, to construct the energy minimised nanotube and nanosheet as well as to calculate their respective atomistic descriptors, the force field was chosen from the list of available ones. Here, the choice of the Sim_LAMMPS_ReaxFF_ChenowethVanDuinGoddard_2008_CHO Force Field for the study of graphane is justified due to its capability to accurately simulate reactive processes and complex bond dynamics [58]. Unlike other force fields such as AIREBO [59], which primarily focus on mechanical properties and static interactions, ReaxFF [60] is designed to handle the chemical reactivity inherent in the formation and transformation of carbon structures. This includes bond formation and breaking, which are crucial for exploring new graphane-based materials and nanostructures such as nanotubes. The ReaxFF force field is highly versatile and has been parameterized to cover a wide range of interactions, enabling it to provide realistic and detailed energy minimizations and structural predictions. Its ability to dynamically adjust to various chemical environments ensures accurate modelling of graphane's behaviour under different conditions, making it an ideal choice for studies aimed at understanding and engineering novel graphane-based nanostructures.

**Fig. 10:**
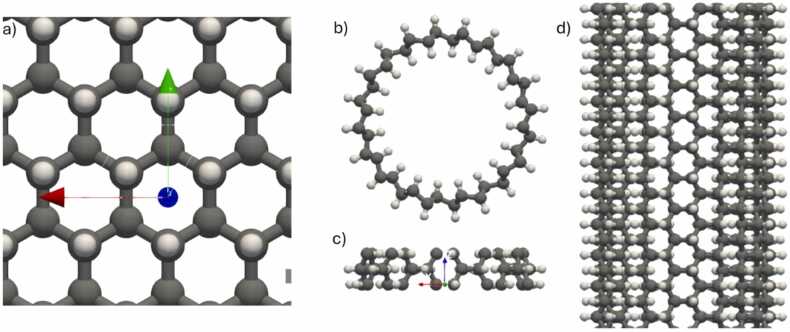
(a) Nanosheet of graphane, (b) projection on xy plane of the energy minimised graphane nanotube with rolling vector (20, 10), (c) the primitive unit cell of graphane Nanotube with rolling vector (20, 10), (d) projection on xz plane of the energy minimised graphane nanotube with rolling vector (20, 10).

After selecting the appropriate force field, the next step involved setting the parameters for energy minimization. The energy tolerance (i.e., the unitless ratio of the energy difference between two consecutive steps to the energy value of the first step) was set to 0.001, the force tolerance (i.e., the 2-norm length of the global 3N-dimensional Force vector consisting of the individual Force vectors of the N atoms of the) to 0.000001 eV/Å, with a maximum of 1000 iterations and 100,000 force/energy evaluations to ensure thorough minimization (see Refs [56], [57] for more details). The minimization process aimed to find the lowest energy configuration for both the graphane nanosheet and the resulting nanotube. The results of the energy minimization together with the key atomistic descriptors for both structures, are estimated and presented in the GUI as shown in Fig. 7. We can see a graphane nanosheet and nanotube in Fig. 10.

**Fig. 11:**
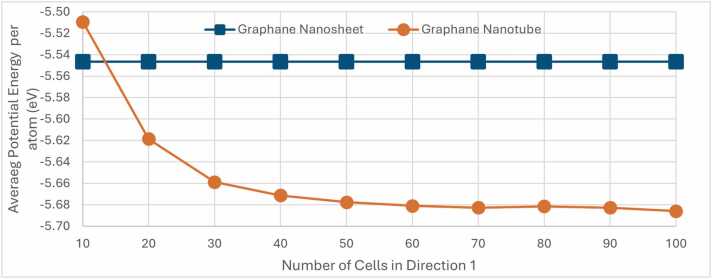
Average potential energy per atom (in eV) for Graphane nanostructures as a function of the number of unit cell replications in Direction 1. The graph compares the average potential energy per atom of a computationally constructed nanosheet (blue dots) with a nanotubes (orange dots).

Fig. 11 presents a detailed comparison of the average potential energy between graphane nanosheets and graphane nanotubes as a function of the number of cells in Direction 1. The graph plots the average potential energy (in eV) on the y-axis against the number of cells in Direction 2 on the *x*-axis.

For the graphane nanosheets, represented by blue dots, the potential energy remains constant across all values of cells in Direction 1 as expected, due to the periodic boundary conditions, consistently hovering around −5.54 eV, which is an extra validation for the accuracy of the calculations conducted by NanoTube Construct.

In contrast, the graphane nanotubes, depicted by orange dots in Fig. 10, exhibit a noticeable decrease in potential energy with an increasing number of unit cell replications in direction 1. Starting from approximately −5.62 eV, the potential energy of the nanotubes progressively decreases and stabilises at around −5.68 eV as the number of unit cell replicates increases. This trend suggests that larger nanotubes, characterised by a greater number of cells in Direction 1, achieve a more energetically stable configuration. This decrease in potential energy with increasing size underscores the enhanced stability of larger graphane nanotubes, likely due to the more favourable atomic arrangements and reduced surface energy effects in these extended structures.

As a second example to showcase the capabilities of the NanoTube Construct web tool, molybdenum disulfide (MoS₂) was chosen due to its unique characteristics and its status as a prominent non-carbon-based nanomaterial. In a similar procedure to graphane, the geometrical construction of the nanosheet and the corresponding nanotube for MoS₂ was performed using the NanoTube Construct. The process began with initialising a single-layer MoS₂ sheet, characterised by its specific atomic lattice structure. The wrap directions were set by specifying the number of unit cell replictaions in two directions, Direction 1 and Direction 2. Various configurations were explored, adjusting the number of unit cell replicates in Direction 1 while keeping Direction 2 constant, to observe how these dimensions affected the nanotube's geometry. The periodicity option is selected again to make the primitive unit cell of the nanotube (see the description of Stage 2 above) which is used to calculate the properties/descriptors for an infinite length nanotube. The tool then wrapped the MoS₂ sheet along the specified directions, and the resulting structure was visualised in 3D. It then calculated the nanotube's geometrical parameters, such as radius and wrap angle (See Table 3).

**Table 3:** Geometrical parameters of MoS_2_ nanotubes constructed using the NanoTube Construction Tool: Number of unit cell replicates in Direction 1 and Direction 2, and the resulting nanotube radius and wrap angle.

Cells in Direction 1	Cells in Direction 2	NanoTube Radius (Å)	Wrap Angle (Degrees)
10	10	4.46	60.00
20	10	8.18	30.00
30	10	13.25	19.11
40	10	18.08	13.90
50	10	22.99	10.89
60	10	27.94	8.95
70	10	32.91	7.59
80	10	37.90	6.59
90	10	42.90	5.82
100	10	47.91	5.21

**Fig. 12:**
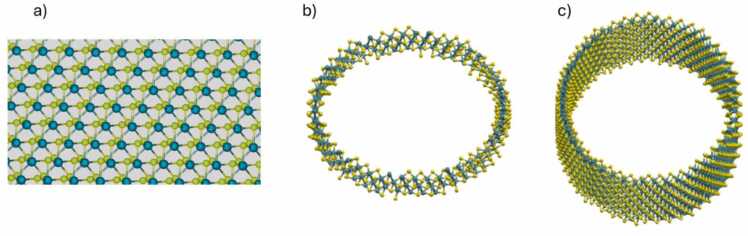
(a) Nanosheet of Molybdenum disulfide, (b) primitive unit cell of the Molybdenum disulfide nanotube with rolling vector (50, 10), (c) Molybdenum disulfide nanotube built by replicating 3 times the primitive unit cell.

The SW_MX2_WenShirodkarPlechac_2017_MoS__MO_201919462778_001 force field was chosen in the context of this study as it was parameterized specifically for MoS₂ by Wen et al.[61], offering a well-established and validated model for MoS_2_ was chosen to examine in the context of this paper. Given its earlier development, this force field has been widely cited and used in the literature, indicating its robustness and reliability in capturing the essential properties of MoS₂. Its parameterization is tailored to accurately represent the covalent interactions and mechanical behaviours of MoS₂, providing a solid foundation for studying its physical characteristics. Additionally, its extensive use in various studies ensures that any potential limitations or strengths are well-documented, offering a reliable reference framework for interpreting simulation results. Then, the same parameters for energy minimization were chosen as in the graphene case. We can see a molybdenum disulphide nanosheet and nanotube in Fig. 12.

**Fig. 13:**
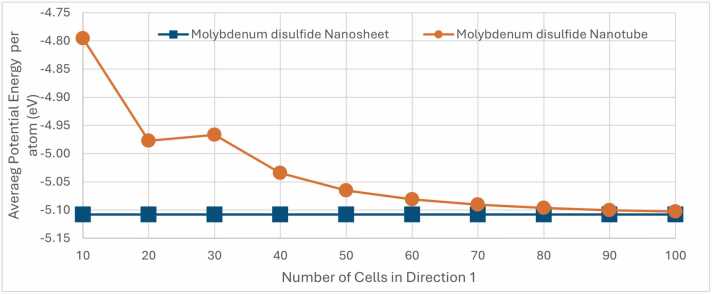
Average potential energy per atom (in eV) for MoS₂ nanostructures as a function of the number of cells in direction 1. The graph compares the potential energy of a nanosheet (blue dots) with nanotubes (orange dots) constructed via NanoTube Construct.

Fig. 13 presents a comparison of the average potential energy between MoS₂ nanosheets and MoS₂ nanotubes as a function of the number of cells in Direction 1. The y-axis represents the average potential energy in eV, while the x-axis shows the number of cells in Direction 1, ranging from 10 to 100. Blue dots represent the potential energy of MoS₂ nanosheets, which remains relatively constant across the range of nanosheet size, at approximately at −5.11 eV. This stability indicates that the energy of the nanosheet does not significantly change with an increasing number of cells in Direction 1. In contrast, the orange dots represent the potential energy of MoS₂ nanotubes, which decreases significantly as the number of cells in Direction 1 increases. Starting around −4.8 eV with 10 cells, the potential energy drops to approximately −5.10 eV at 100 cells. This trend suggests that larger MoS₂ nanotubes, achieve a more energetically stable configuration and are in agreement with experimental measurements [26].

## 5 Conclusions

The development of NanoTube Construct presented in this paper represents a significant advance in the computational design and construction of nanotubes from single-layer materials. This tool addresses critical limitations of existing nanotube construction methods by enabling the creation of nanotubes with non-zero thickness and various symmetries. Its integration into the Enalos Cloud Platform ensures accessibility to a broader range of researchers and users, facilitating the exploration of diverse NMs beyond traditional carbon-based structures.

The Nanotube Construct’s capability to predict stable atomic configurations is particularly valuable for guiding experimental synthesis, thereby reducing the trial-and-error associated with materials discovery. By providing insights into the stability and properties of novel nanotube structures, the NanoTube Construct aids in the rational design of NMs with tailored properties for specific applications in electronics, energy storage, and biomedical fields.

Moreover, the inclusion of atomistic descriptors such as coordination number and potential energy as inputs for machine learning models opens new avenues for predicting material properties like toxicity and reactivity. This integration of computational tools for virtual NMs creation and exploration with predictive modelling of the properties and interactions of the resulting NMs enhances the design of safer and more effective NMs, aligning with the principles of the SSbD framework.

The practical examples of constructing nanotubes from graphane and MoS₂ demonstrate the tool’s versatility and robustness. The energy minimisation and structural stability analysis comparison of NanoTube Construct predictions with experimental measurements [26] confirm the reliability of the NanoTube Construct in producing realistic and stable nanotube configurations and stability predictions. This capability significantly contributes to advancing nanoscale and advanced materials research by providing a comprehensive and user-friendly platform for the construction and analysis of a wide range of nanotube structures.

## Funding

This project has received funding under the European Union’s Horizon 2020 research and Innovation programme via DIAGONAL under grant agreement no. 953152 and the European Union’s H2020 Marie Skłodowska-Curie Actions via CompSafeNano under grant agreement no. 101008099.

## CRediT authorship contribution statement

**Panagiotis D.Kolokathis**: Conceptualization, Methodology, Software, Formal analysis, Writing — original draft, Visualization. **Dimitrios Zouraris**: Validation, Investigation, Writing — original draft, Visualization. **Nikolaos K. Sidiropoulos**: Software. **Andreas Tsoumanis**: Software, Writing — review and editing. **Georgia Melagraki**: Writing — review and editing. **Iseult Lynch:** Validation, Writing — review and editing, Supervision, Funding acquisition. **Antreas Afantitis**:Conceptualization, Writing—review and editing, Supervision, Project administration, Funding acquisition.

## Declaration of Competing Interest

PK, DZ, NS, AT and AA are employed by NovaMechanics, a cheminformatics and materials informatics company.
